# Enhancing Wildlife Monitoring: An Advanced AI Approach for Accurate Giant Panda Behavior Detection and Conservation Insights

**DOI:** 10.3390/ani16060943

**Published:** 2026-03-17

**Authors:** Jin Hou, Chaoyu Liu, Dan Liu, Vanessa Hull, Yutong Wang, Xinyi Zhao, Yingchun Tan, Xiaogang Shi, Yuehong Cheng, Zhuo Tang, Desheng Li, Jifeng Ning, Jindong Zhang

**Affiliations:** 1 Key Laboratory of Southwest China Wildlife Resources Conservation (Ministry of Education), China West Normal University, Nanchong 637002, China; houj815@163.com (J.H.);; 2 College of Life Sciences, Beijing Normal University, Beijing 100875, China; 3 Nicholas School of Environment, Duke University, Durham, NC 27705, USA; 4 College of Information Engineering, Northwest A&F University, Yangling 712100, China; 5 M3-BIORES-Measure, Model & Manage Bioresponses, KU Leuven, Kasteelpark Arenberg 30, B-3001 Leuven, Belgium; 6 Department of Wildlife Ecology and Conservation, University of Florida, Gainesville, FL 32611, USA; 7 Wolong National Nature Reserve, Wenchuan 623006, China; 8 China Conservation and Research Centre for the Giant Panda, Key Laboratory of State Forestry and Grassland Administration on the Giant Panda, Chengdu 610057, China

**Keywords:** behavior detection networks, deep learning, giant panda, infrared camera

## Abstract

As global demands for nature reserve management intensify, intelligent monitoring has become a pivotal trend. Integrating artificial intelligence with infrared camera traps enables automated analysis of endangered species behavior, providing timely insights for conservation. However, complex habitats often degrade the performance of existing detection technologies. Focusing on the giant panda—a flagship conservation species—we constructed a novel dataset from long-term field monitoring videos and developed an improved PandaSlowFast network. Our model employs channel attention to enhance temporal features, uses small-kernel depth-wise convolutions and dilated convolutions to expand receptive fields for spatial feature extraction, and introduces the Adaptive SwisH activation function to improve adaptability and training stability. The results show that PandaSlowFast achieves 85.38% mean average precision (mAP), outperforming existing methods. An FP16-quantized version maintains comparable accuracy (85.16% mAP) while running at 3.2 frames per second on a Raspberry Pi 4, demonstrating practical deployability for on-site monitoring. This work provides technical support for intelligent panda behavior analysis and offers a transferable methodology for monitoring other rare species, contributing to biodiversity conservation.

## Simple Summary

Protecting endangered species like giant pandas requires constant monitoring, but current methods often struggle with complex forest environments. This study introduces PandaSlowFast, an improved AI model that automatically recognizes wild panda behaviors from video footage. By enhancing how the model processes motion patterns and fine image details, we achieved 85.38% accuracy on a new dataset of long-term monitoring videos—significantly outperforming existing approaches. A compact version also ran effectively on a simple edge device like Raspberry Pi, making it practical for on-site deployment. This technology supports real-time wildlife monitoring and can be adapted for other rare species.

## 1. Introduction

In the context of accelerating global biodiversity loss, wildlife conservation has become an urgent ecological and societal priority. Behavioral monitoring is a core component of conservation biology, providing critical insights into species’ survival strategies, population dynamics, and interactions with their environments [1]. Observations of behaviors such as foraging, movement, and breeding help reveal responses to environmental change and emerging ecological pressures, while long-term behavioral shifts can serve as early indicators of population decline and ecosystem imbalance [2,3]. These insights are essential for evidence-based conservation planning and adaptive management.

Infrared camera trap technology is now widely used for non-invasive, continuous wildlife monitoring, generating massive volumes of video data that authentically capture animal behavioral patterns across different seasons and habitats [4]. However, the sheer scale, inherent redundancy, and visual complexity of these data pose significant analytical challenges. Manual video inspection is time-consuming and labor-intensive, with particularly low efficiency in heterogeneous natural environments where behaviors are subtle, overlapping, and context-dependent [5]. Furthermore, variations in illumination, background clutter, and animal appearance complicate reliable interpretation, highlighting the urgent need for more advanced, automated, and scalable analytical methods [6]. In recent years, advances in deep learning have provided new pathways for intelligent wildlife video analysis [7,8]. Although deep learning has been successfully applied to tasks such as species identification and behavior classification [9,10], most existing studies focus on behavior recognition rather than the more challenging task of behavior detection. Behavior detection requires simultaneously identifying the behavioral category, temporal interval, and spatial location within continuous video streams [11,12]. The rich spatiotemporal information derived from detection is crucial for quantifying key ecological metrics such as behavioral duration, transition patterns, and individual interactions.

While deep learning has been successfully applied to wildlife identification and behavior classification [9,10], most existing studies focus on behavior recognition rather than behavior detection. Behavior detection is substantially more challenging, as it requires simultaneously identifying behavior categories, temporal intervals, and spatial locations within continuous video streams [11]. Nevertheless, this richer spatiotemporal information is essential for quantifying behavioral duration, transitions, and interactions, which are critical for ecological inference.

The giant panda (*Ailuropoda melanoleuca*) is an ideal focus due to its dual role as a global conservation flagship and ecological indicator species [13,14]. As a flagship species, protecting pandas and their habitats safeguards numerous sympatric species, creating a significant conservation multiplier effect. As an ecological indicator, behavioral changes in pandas—such as altered movement patterns—provide sensitive early warnings of environmental pressures, often manifesting before detectable population declines [15]. The need for intelligent behavior analysis stems from both ecological requirements and technological gaps. Extensive infrared camera networks generate thousands of hours of footage annually, but the lack of effective automated tools limits data utilization, as manual analysis is prohibitively time-consuming. Early CNN-based methods suffered from limited temporal modeling [16]; Transformer-based approaches improved spatiotemporal feature learning but focused on segment-level classification rather than precise localization [17]. Recent SlowFast-based efforts have begun addressing behavior detection in continuous videos, but their accuracy remains insufficient for large-scale field deployment [18]. Behavior recognition and behavior detection represent fundamentally different tasks. While recognition identifies which behavior occurs, detection determines when and where behaviors take place, enabling detailed analysis of behavioral sequences, durations, and interactions [19,20]. For wildlife monitoring in natural environments, behavior detection is therefore more closely aligned with practical conservation requirements.

In summary, despite notable progress, existing research on giant panda behavior analysis remains limited by a focus on captive settings and classification-based methods. The convergence of urgent conservation needs, underutilized monitoring data, and recent advances in video understanding creates both the motivation and the opportunity for this study. There is a clear need for robust behavior detection frameworks capable of handling complex, real-world infrared video data to support fine-grained ecological analysis and informed conservation decision-making.

To address these issues, we developed a new behavior detection model using giant pandas as a case study, integrating deep learning with infrared camera data. The major contributions of our research include:

(1) Establishing a frame-by-frame annotated dataset for wild panda behavior detection based on long-term monitoring data, covering diverse behaviors in various natural scenarios with rigorous annotation for accuracy and reliability.

(2) Proposing a wild panda behavior detection model (PandaSlowFast) that improves upon the SlowFast algorithm by using a Temporal Attention Unit (TAU) to enhance spatiotemporal feature sensitivity and representation, and introduces the Adaptive SwisH activation function to improve model adaptability and contextual awareness.

(3) Validating the method using a dataset of wild giant panda behavior detection and evaluating its effectiveness compared to existing behavior detection algorithm models.

This study not only advances panda conservation but also contributes to developing intelligent wildlife monitoring systems, providing a technical foundation and research perspective for conserving globally rare and endangered species.

## 2. Materials and Methods

### 2.1. Data Collection

**Figure 1:**
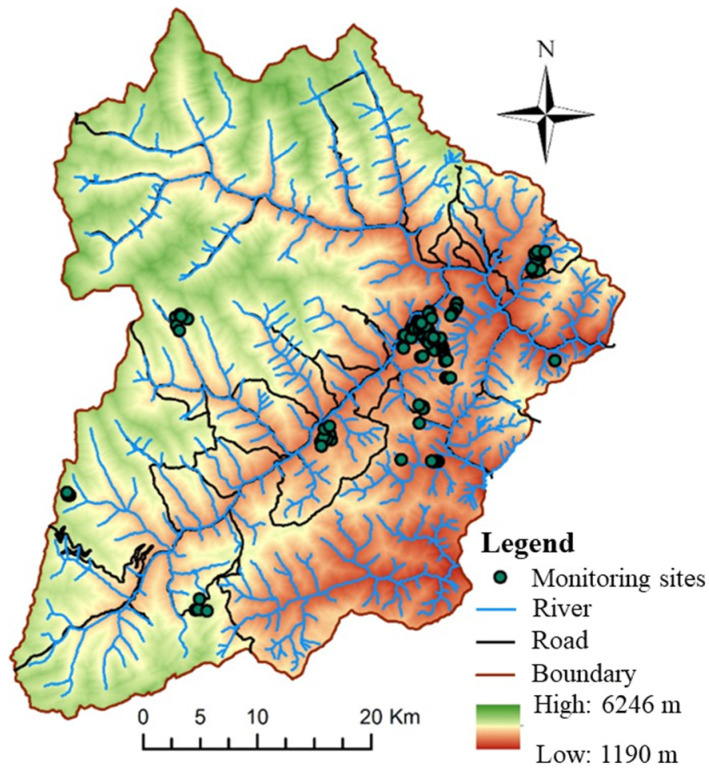
**Monitoring locations of infrared cameras for giant pandas in the Wolong area of the Giant Panda National Park in China.**

This study was conducted in the Wolong area of the Giant Panda National Park in China, a high mountain valley region covering approximately 2000 km^2^ with elevations from 1150 to 6250 m. The area features rich vertical zonal vegetation and hosts over 4000 plant species and 2000 animal species, including many ancient relic and endemic taxa [21]. According to the most recent national survey, over 100 wild giant pandas inhabit the Wolong area [22]. Based on molecular analysis and field monitoring records, at least 20 individual pandas were included in our behavioral sampling. From 2018 to 2022, approximately 150 infrared cameras were deployed in panda-frequented areas—prioritizing bamboo-rich zones, gentle slopes, and river–valley corridors—following the long-term monitoring principles of the Wolong National Nature Reserve [23]. The resulting spatial clustering of sampling points (Figure 1) reflects the species’ actual habitat-use distribution rather than deployment bias. Habitat analysis reveals that pandas prefer coniferous–broadleaf mixed forests at 1600–3400 m elevation, with gentle slopes (<5°), access to water within 1000 m, abundant staple bamboo, and low shrub density, while clearly avoiding areas within 3500 m of human settlements. These habitat characteristics provide important references for panda conservation and corridor planning. As shown in Figure 1, approximately 150 infrared cameras were deployed in the region from 2018 to 2022.

The camera placement in this study followed the principles of the Wolong National Nature Reserve’s long-term monitoring system, prioritizing areas of intensive giant panda activity, including bamboo-rich zones, gentle slopes, and river–valley corridors. Consequently, the sampling points in Figure 1 exhibit spatial clustering. This pattern reflects the species’ actual habitat-use distribution rather than any arbitrary bias in camera deployment.

### 2.2. Preprocessing and Annotation of Panda Behavior Data

**Figure 2:**
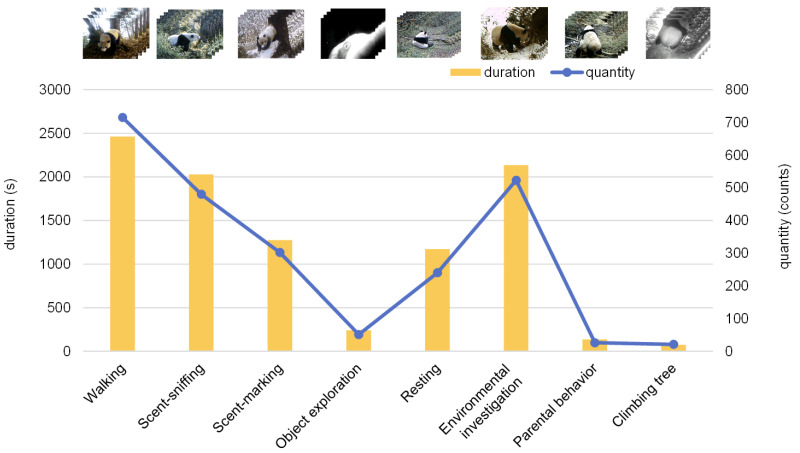
**Duration of giant panda behaviors and number of video segments in the dataset.**

Using four years of field monitoring data, we collected 984 videos of wild giant panda behaviors. After quality filtering, 547 videos were segmented into 1879 clips of 10 s, resulting in a total duration of 5.2 h. Given the challenges of infrared monitoring in the wild, we applied strict quality control during data selection and annotation to remove low-quality clips, resolve ambiguous cases through cross-validation, and maintain behavioral diversity under varied environmental conditions. To the best of our knowledge, this is the first dataset for wild giant pandas with frame-level behavioral bounding box annotations, comprising 14,427 bounding boxes. In terms of both scale and annotation granularity, it represents a relatively large dataset for fine-grained behavior detection in wild animals. We split videos into 10 s segments for management, but all behaviors are annotated at the frame level, so multiple behaviors within a segment are still distinguished from one another. This segmentation reflects typical infrared camera recordings and helps remove irrelevant frames, without forcing behaviors to last 10 s or reducing classification accuracy. We defined and classified the behavior data based on categories outlined in previous research [24,25,26] (see Table 1). In the annotation process, we explicitly accounted for the spatiotemporal overlap commonly observed in wild animal behaviors. For instance, in scenarios where “walking” and “parental behavior” occur concurrently, each behavior is independently annotated using a multi-label scheme, with separate temporal segments and spatial bounding boxes assigned to each. This annotation strategy ensures that the training data captures the real-world complexity of co-occurring behaviors, thereby enabling the model to learn and accurately infer such intricate spatiotemporal behavior patterns. To address the unique characteristics of “parental behavior” as a multi-individual interactive activity, we implemented strict annotation criteria. This category is labeled only when clear and discernible interactions between a panda mother and cub (e.g., nursing, carrying) are visible in the video. Following classification, various types of behavior data were organized, as illustrated in Figure 2. The orange bars represent the duration of behaviors, indicating that walking, environmental investigation, and scent-sniffing behaviors of giant pandas in the wild tend to last longer than other behaviors. The blue bars correspond to the number of videos capturing these behaviors, with a greater number of videos recorded for behaviors that exhibit longer durations.

**Figure 3:**
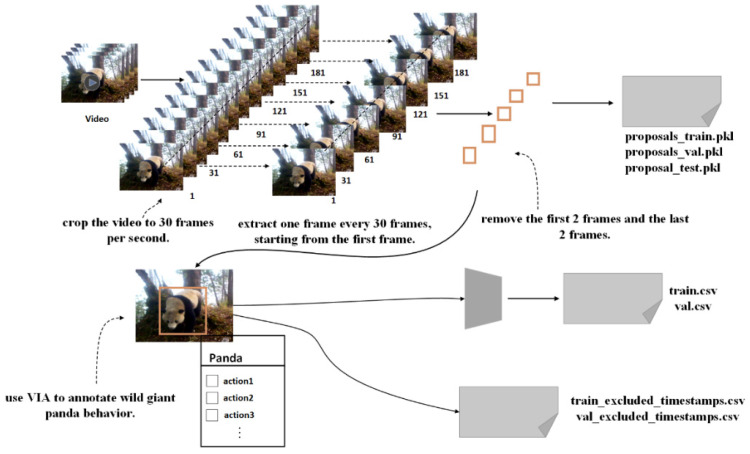
**Flowchart for the creation of the giant panda field behavior detection dataset.**

Following the completion of the overall classification of the data, we proceeded to further compile the dataset, as illustrated in Figure 3. We first cropped the videos to extract 30 frames per second. Starting from the first frame, we selected every 30th frame while removing the first two frames and the last two frames. The first and last two frames were removed because they often contain incomplete or overlapping actions, motion blur, or occlusion, which increase labeling ambiguity. Excluding them ensures the retained frames better represent the stable phase of the behavior, improving annotation quality. This process resulted in the extraction of a pkl file containing temporal information. Subsequently, the dataset underwent frame-by-frame annotation, which was divided into two parts: behavior annotation and bounding box annotation. The behavior annotation involved describing the actions exhibited by the giant pandas in the video frames, while the bounding box annotation described the spatial location information of the pandas. The standard for bounding box annotation was defined as the smallest rectangle that could enclose the giant panda. We utilized the VGG Image Annotator to annotate the behaviors of the wild giant pandas, resulting in a CSV file containing the behavior information of the pandas.

**Figure 4:**
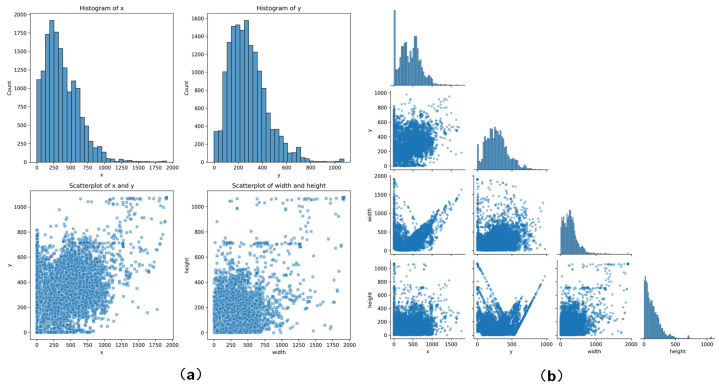
**Spatial position visualization of giant panda behavior bounding boxes.** (**a**) The histograms illustrate the frequency distributions of x and y, while the scatter plots depict the distribution relationships between the pairs (x, y) and (width, height). The use of transparent small squares intuitively reflects the density of the data. (**b**) This section presents the pairwise relationships among the four variables: x, y, width, and height. It includes histograms of the variables themselves and scatter plots between the variables, revealing the distribution characteristics and correlations from a global perspective.

Overall, the annotation of the giant panda behavior dataset was completed by 30 professional volunteers over a period of three months. To ensure data quality, we implemented unified training, independent cross-annotation with discussion-based reconciliation, and a dedicated review of ambiguous segments, thereby minimizing annotation bias and ensuring data reliability, resulting in the annotation of 14,427 behavior bounding boxes. The spatial distribution, width distribution, and height distribution of the annotated giant panda behavior bounding boxes are illustrated in Figure 3. From the perspective of the spatial distribution of the giant panda behavior bounding boxes within the frames, it is evident that their distribution exhibits substantial diversity and richness. Some bounding boxes occupy relatively small areas of the frame, indicating that the giant pandas may be in a more confined activity space or in specific behavioral states. Conversely, some bounding boxes cover larger areas of the frame, which may be associated with active movement, exploratory behaviors, or interactions with other individuals. Fixed camera positioning, shooting distance, and perspective distortion may affect bounding-box size, but the impact of these factors is limited due to standardized deployment ensuring consistent viewpoints. The model learns temporal and morphological features rather than relying on absolute box size, and preprocessing with scale normalization and random resizing enhances robustness. This variation in spatial distribution reveals the diversity of giant panda behavior patterns, reflecting the realities of field monitoring (Figure 4a,b).

The dataset was partitioned by camera location and recording time rather than using a random process to allow for greater independence between samples. Video clips from the same camera were preferentially assigned to a single subset to reduce scene overlap, and different time periods were selected to avoid adjacent frames being split across sets. The final division of training, validation, and test sets in a ratio of 7:1:2 ensured a balanced distribution of behavior categories while minimizing both spatial and temporal overlap between subsets, enabling a more reliable assessment of the model’s generalizability.

### 2.3. Construction and Improvement of the Giant Panda Behavior Detection Model

**Figure 5:**
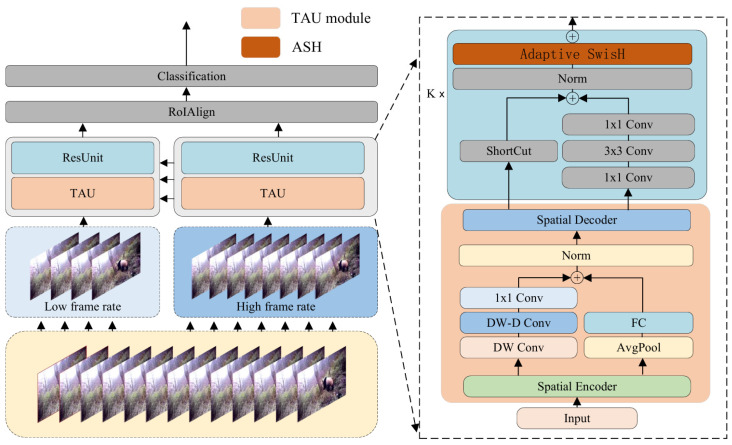
**Overall network structure of PandaSlowFast. The left side shows the overall structure of PandaSlowFast, while the right side illustrates the internal components of the core module.**

This study proposes a method for detecting the behavior of wild giant pandas based on the SlowFast network [27], which we have named PandaSlowFast. The overall structure is illustrated in Figure 5. As shown in the figure, we embed the TAU attention mechanism into the important convolutions of the convolutional blocks, further enhancing the weight of feature extraction and improving the network’s ability to extract and represent behavioral features. Additionally, we introduce the ASH (Adaptive SwisH) activation function to dynamically adjust the activation states, enhancing the model’s robustness and nonlinear expression capability when handling complex features.

This study adopts the standard SlowFast dual-rate sampling strategy to achieve a complementary balance between semantic information and motion features. The Slow pathway primarily captures high-level semantic information in the video, which can be obtained from single frames or sparsely sampled frames; therefore, it operates at a lower frame rate. The sampling interval is denoted as τ, meaning that only one frame out of every τ frames is processed, with τ typically set to 16. For a 30 fps video, the Slow pathway samples approximately 2 frames per second, enabling long-term semantic modeling while avoiding temporal redundancy. Running in parallel with the Slow pathway, the Fast pathway is a lightweight 3D convolutional model designed to capture fine-grained motion features and behavioral dynamics. It operates at a higher temporal resolution with a sampling interval of τ/α, where α > 1. In this study, α is set to 8, resulting in approximately 15 frames per second for a 30 fps video, thereby enhancing sensitivity to short-term motion. The original dataset fully retains the native 30 fps frame rate, and τ-based sampling is applied only at the model input stage. Each 10 s video clip still contains about 300 frames, from which the model selectively samples as needed, avoiding information loss while reducing redundancy.

#### 2.3.1. Spatiotemporal Attention Unit

**Figure 6:**
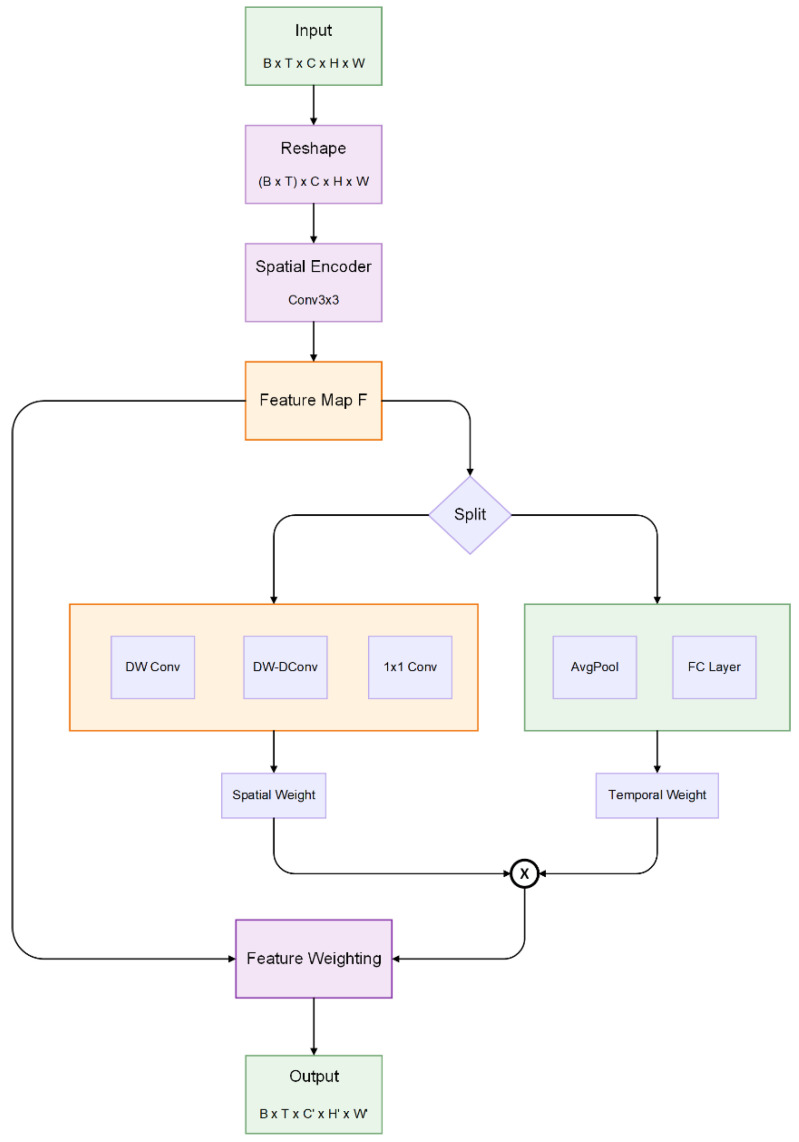
**The structure of the Temporal Attention Unit.**

Traditional methods typically rely on recurrent neural networks (RNNs) to model temporal dependencies [28,29,30,31]. However, RNNs have inherent limitations, including low computational efficiency, difficulty in capturing long-term dependencies, and susceptibility to issues such as vanishing or exploding gradients. Inspired by the work of [32], we improve the SlowFast behavior detection network by employing a model known as the TAU (Temporal Attention Unit), which does not utilize recurrent neural networks but instead harnesses an attention mechanism to process temporal evolution in parallel. The structure of the TAU is illustrated in Figure 6.

Assuming an input batch of video tensors β ∈ R^(B × T × C × H × W)^, where the number of videos B = |β| is known, T represents the past T frames, C denotes the number of channels in the video frames, and H and W are the width and height of the video frames, respectively. In the spatial encoder and decoder, we reshape the sequential input data B × T × C × H × W to (B × T) × C × H × W to focus solely on spatial correlations. In the temporal module, we reshape the features from B × T × C × H × W to B × (T × C) × H × W, allowing the frames to be arranged sequentially along the channel dimension (Figure 6).

**Figure 7:**
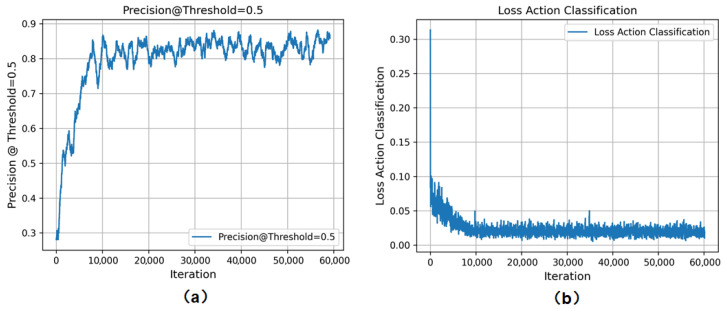
(**a**) **Accuracy of PandaSlowFast**; (**b**) **loss curves of PandaSlowFast**.

The TAU decomposes spatiotemporal attention into two components: intra-frame static attention and inter-frame dynamic attention, as illustrated in Figure 7. Intra-frame static attention employs small kernel depth-wise convolutions and depth-wise dilated convolutions to obtain large receptive field, enabling it to capture long-distance dependencies within a single frame. Inter-frame dynamic attention utilizes channel-wise attention mechanisms to learn the channel weights across different frames, thus capturing the trends of changes between frames. The definitions of the TAU attention unit’s intra-frame static attention (SA) and inter-frame dynamic attention (DA) are given by the following Equations (1)–(3).

(1) $$\;SA=Conv_{1\times 1}\left(DW-D\;Con\left(DW\;Conv\left(H\right)\right)\right)$$

(2) $$\;DA=FC\left(AvgPool\left(H\right)\right)$$

(3) $$H^{\prime }=\left(SA⊗DA\right)⊙H$$

#### 2.3.2. Adaptive SwisH Activation Function

Through our experiments, we found that although the ReLU activation function used in the original SlowFast network efficiently facilitates gradient descent and backpropagation while avoiding issues of gradient explosion and vanishing gradients, it does not effectively utilize contextual information to determine the values of element spatiotemporal behavior detection. While parameterized activation functions update their parameters during training, the obtained parameters remain fixed during the inference phase. In light of this, we introduce the ASH (Adaptive SwisH) activation function [33] to replace the ReLU activation function. The ASH activation function employs an adaptive threshold to correct the input, taking into account the entire context of the input. Unlike existing activation functions, which have limitations in representing the variability of threshold potentials based on the location and type of individual neurons in human brain function, the ASH activation function aims to address this variability. The principle of the ASH activation function is as follows:

Assume X is the input to the ASH activation function, where (x^(i)^ \in X) denotes the (i)-th element of the input. The ASH activation function samples the top (k%) of the input, represented as follows:

(4) $$f\left(x^{i}\right)=\left\{\begin{array}{cc} x^{\left(i\right)}, & \;\;if\;{\;x}^{\left(i\right)}\;\;\geq \;\mu X\;+\;z_{k}\sigma X\\ 0\;\;\;\;, & \;\;otherwise \end{array}\right.$$

where μX and σX denote the mean and standard deviation of all elements in X, respectively, and $z_{k}$ represents the Z-score corresponding to the sample percentile k. For simplicity, we assume the Heaviside step function is defined as $H\left(x\right)=\frac{d}{dx}max(0,x)$. Thus, Equation (4) can be expressed as follows:

(5) $$f\left(x^{i}\right)=\;x^{i}H\left(x^{i}-\mu x-z_{k}\sigma x\right)$$

The Heavis step function is analytically approximated by $2H\left(x\right)=1+1\tanh\left(\alpha x\right)\;$ [34]. Therefore, the ASH activation function can be expressed as follows:

(6) $$\begin{array}{cc} f\left(x^{\left(i\right)}\right) & =x^{\left(i\right)}H\left(x^{\left(i\right)}-\mu x-z_{k}\sigma x\right)\\ & =\frac{1}{2}x^{\left(i\right)}+\frac{1}{2}x^{\left(i\right)}tan\;h\left(\alpha x^{\left(i\right)}-\mu x-z_{k}\sigma x\right)\;\;\\ & =\frac{x^{\left(i\right)}}{1+e^{-2\alpha \left(x^{\left(i\right)}-\mu x-z_{k}\sigma x\right)}}\; \end{array}$$

Since $z_{k}$ is arranged in an arithmetic sequence, $z_{k}$ represents the sampling percentiles, enabling the ASH activation function to possess trainability parameterization characteristics. By optimizing the value of $z_{k}$, the ASH activation function can exhibit different threshold behaviors. Therefore, based on random sampling the input and these varied, the ASH activation function is capable of demonstrating diverse activation states, a feature similar to the behavior of human neurons, synapses, and their electrical activities.

### 2.4. Behavioral Detection Model Experiments and Evaluation

#### 2.4.1. Parameter Settings and Experimental Environment

During the training process, we employed operations such as random scaling, cropping, and flipping to enhance data diversity. We utilized resample rate, speed ratio, and channel ratio to handle features with varying speed channels, adapting to various data characteristics. The weight of positive samples was set to 1 to adjust the sample weights and address the issue of class imbalance. The SGD optimizer, combined with an appropriate learning rate, momentum, and weight decay, facilitated the model’s convergence to a better solution. We adopted a learning rate scheduling strategy that combines LinearLR and CosineAnnealingLR, allowing for rapid adaptation during the initial training stages and slower convergence in later stages, thereby improving training efficacy. The initial learning rate was set to 0.075, with momentum at 0.9 and weight decay at (10^−5^). We controlled the settings for the automatic scaling of the learning rate, deciding whether to enable this feature based on actual conditions. Meanwhile, the behavior detection task is formulated as a multi-label learning problem, as multiple behaviors may occur within the same video clip. Accordingly, a sigmoid activation function with binary cross-entropy loss is adopted for classification. Each video clip is treated as an independent training sample and directly associated with its corresponding behavior labels, without introducing anchor-based or positive/negative sample matching mechanisms. The classification head follows a lightweight design: features from different temporal pathways are first pooled and concatenated, then fused through a 1 × 1 × 1 convolution, followed by global average pooling and a fully connected layer to produce behavior predictions. A single classification loss with a weight of 1 is used during training, and no auxiliary losses or re-weighting strategies are introduced to maintain training stability. In addition, we established a base batch size for training. This study conducted experiments using four 2080Ti GPUs, totaling 48 GB of memory, with a batch size of 64, and a total of 50 training epochs.

#### 2.4.2. Model Validation and Evaluation

To evaluate the algorithm’s performance, this study employs the following metrics: average precision (AP), mean average precision (mAP), and the number of network parameters. AP represents the average Precision at different Recall values and serves as a comprehensive measure of model performance. mAP is the average of AP across multiple categories and is an important metric for assessing the performance of multi-class classifiers. The number of network parameters indicates the complexity of the behavioral detection network, encompassing the values of network weights and biases, which are used to compute the mapping relationship between input and output data. It is a crucial indicator of model complexity; the higher the number of parameters, the more complex the model, resulting in longer computation times.

To reflect the experimental effects of our model, we trained on the training set and reported baseline performance on the test set. Additionally, we conducted comparative analysis with the earliest teams that explored captive giant pandas, as well as with recent advanced backbone network algorithms based on the Transformer architecture. The training settings for the six behavior recognition methods were uniformly set to the default parameter values as specified in the original papers. Moreover, to validate the effectiveness of each improvement made to the SlowFast network, we performed ablation experiments. We evaluated the independent and combined effects of adding the Adaptive SwisH activation function and the temporal attention module. Finally, to analyze the interrelationships between different categories and identify which categories are prone to confusion, we visualized the confusion matrix for each benchmark model.

## 3. Results

### 3.1. Experimental Effects of the PandaSlowFast Behavior Detection Model

The experimental results indicate that the accuracy and loss curves of our algorithm have both converged. The proposed improved PandaSlowFast network maintains a relatively high accuracy level with a false positive rate of approximately 0.5. At this point, the accuracy and recall rates are well-balanced, demonstrating an overall effective performance (Figure 7).

**Figure 8:**
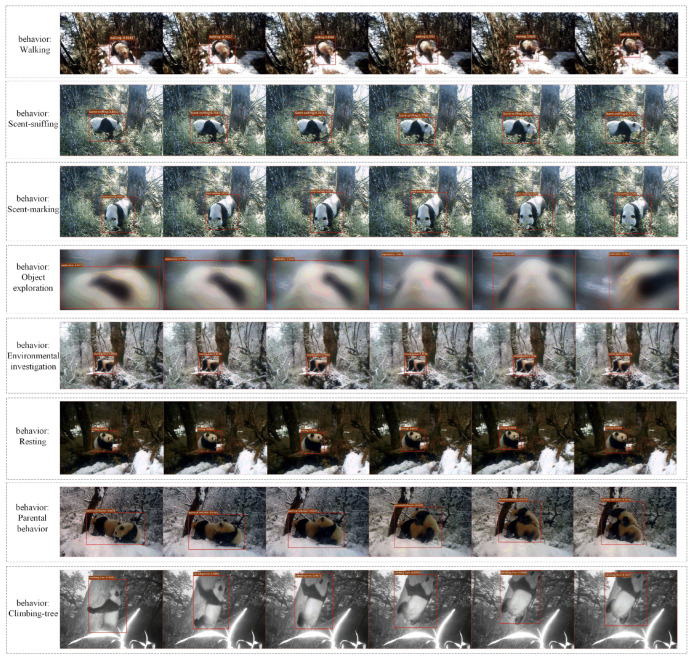
**Visualization of behavior detection results.**

**Figure 9:**
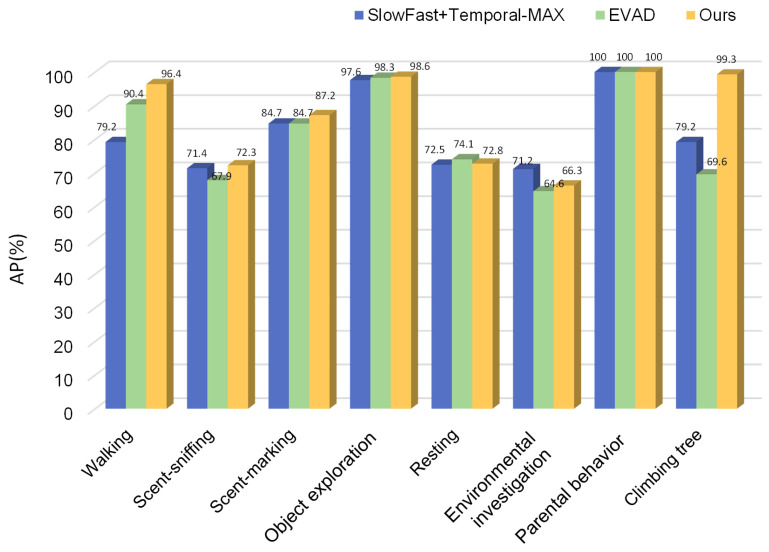
**Bar chart comparing AP of different algorithms for each behavior category.**

We further conducted a multi-perspective qualitative analysis of giant panda behavior detection, extracting six frames from each video for visualization. The detection boxes accurately locate the body parts of the giant pandas, with the model precisely capturing the contact points between the pandas’ heads and the target points. For walking behaviors, which exhibit significant dynamic changes, the detection boxes successfully track the movements. Even under conditions where the subject is captured at night or partially occluded, the model remains effective in detecting the target (as illustrated by the object exploration behavior in Figure 8). The results demonstrate that our algorithm exhibits excellent performance in detecting wild giant panda behaviors, accurately identifying the correct behavior categories from complete video sequences across various environmental backgrounds and shooting angles (Figure 9).

Comparing the PandaSlowFast network with several other mainstream behavior detection methods, we found that this approach outperforms earlier methods proposed by teams exploring captive giant panda behavior detection by 39.17% [35]. In comparison to the baseline SlowFast model, there is a 7.72% improvement, and when compared to the SlowFast model with Temporal-Max Pooling, there is a 2.22% enhancement [36]. Furthermore, in contrast to the currently popular behavior recognition algorithms based on the Transformer architecture, our network offers advantages of smaller parameter size and higher recognition accuracy, making it more suitable for small sample learning with infrared camera monitoring data. Specifically, our method achieves a 2.70% performance improvement over the Transformer-based EVAD model [32] and an 8.23% enhancement compared to the VideoMAE algorithm [37] (Table 2).

To further analyze the performance of different models across various behavior categories, we evaluated the accuracy of the top three algorithms with the highest average accuracy for each behavior type (Figure 9). The results indicate that the PandaSlowFast network, which is based on the TAU attention unit, shows varying degrees of improvement in recognition accuracy for multiple behaviors. Specifically, the recognition accuracies for walking, scent-sniffing, scent-marking, object exploration, and climbing tree behaviors surpassed those of the other two algorithms. Furthermore, the model achieved a remarkable 100% recognition accuracy for parental behavior, underscoring the effectiveness of the model developed in this study (Figure 9).

### 3.2. Ablation Experiment Analysis

For the wild giant panda behavior detection model, the introduction of the Adaptive SwisH activation function alone improved the model’s mAP by 0.80%, reaching 83.95%. This indicates that the activation function offers certain advantages in enhancing the model’s nonlinear expressive capabilities. After incorporating the temporal attention module, the mAP increased to 85.16% (an improvement of 2.01%), demonstrating that this module effectively captures the spatiotemporal dependencies in behavior detection, enhancing the sensitivity of features to both time and space. When both modules were integrated, the mAP further increased to 85.37% (an additional improvement of 2.22%), indicating a synergistic gain in model performance optimization from the combination of the two modules (Table 3).

To validate the effectiveness of the dual-rate sampling strategy in PandaSlowFast, we designed an ablation study by varying the temporal sampling intervals of the Slow and Fast pathways. Specifically, we compared a single-path variant (SlowOnly) with multiple dual-path configurations (β = 1/4, β = 1/6, β = 1/8, β = 1/12, and β = 1/16). The models were evaluated in terms of mean average precision (mAP), fine-grained behavior recognition accuracy, and computational efficiency. As shown in Table 4, overly sparse sampling led to a loss of temporal information and performance degradation, whereas overly dense sampling introduced significant computational overhead with only marginal accuracy gains. The results indicate that β = 1/8 provides the optimal trade-off between detection accuracy and efficiency.

To ensure the statistical robustness of the reported performance gains, we conducted three independent training runs with different random seeds for the baseline SlowFast model and three proposed variants. As summarized in Table 5, PandaSlowFast achieves an average mAP of 85.38% on the test set with a low standard deviation of ±0.04%, indicating both stable and consistent performance improvements. A paired *t*-test further confirms that the mAP gain of PandaSlowFast over the baseline model is statistically significant (*p* = 0.002 < 0.01), suggesting that the observed improvements are unlikely to be attributed to random variation.

To explicitly evaluate the capability of the TAU module in modeling long-term temporal dependencies, we categorized behaviors in the test set according to their temporal duration into short-duration behaviors (<2 s, e.g., climbing tree) and long-duration behaviors (>5 s, e.g., resting and walking). As shown in Table 6, the introduction of the TAU module leads to a substantially larger performance improvement for long-duration behaviors (+3.2% mAP) compared to short-duration behaviors (+0.9% mAP). This clear discrepancy provides strong evidence that TAU effectively enhances the modeling of long-range temporal dependencies through its frame-wise dynamic attention mechanism, rather than yielding uniform gains across all behavior types.

**Figure 10:**
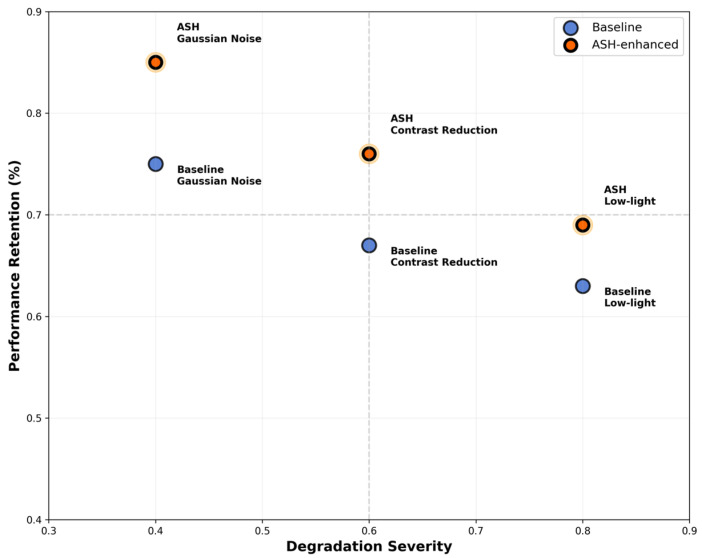
**Performance retention of ASH-enhanced model versus baseline under controlled image degradation.** The *x*-axis represents the severity of image degradation (ranging from mild noise to severe low-light conditions); the *y*-axis denotes the model’s performance retention rate (the ratio of degraded mAP to the original mAP).

To evaluate the effectiveness of ASH under complex real-world field conditions, we applied three controlled image degradations to the test videos: Gaussian noise (σ = 25), contrast reduction (50%), and brightness reduction (40%). As illustrated in Figure 10, the model incorporating the ASH activation function consistently demonstrates superior performance retention across all degradation scenarios. In particular, under low-light conditions, the mAP of the ASH-enhanced model decreases by only 4.1%, compared with a 7.8% reduction for the baseline model. These results indicate that ASH’s adaptive thresholding mechanism more effectively activates robust features that remain informative under adverse imaging conditions.

### 3.3. Confusion Matrix Analysis of Mainstream Models

**Figure 11:**
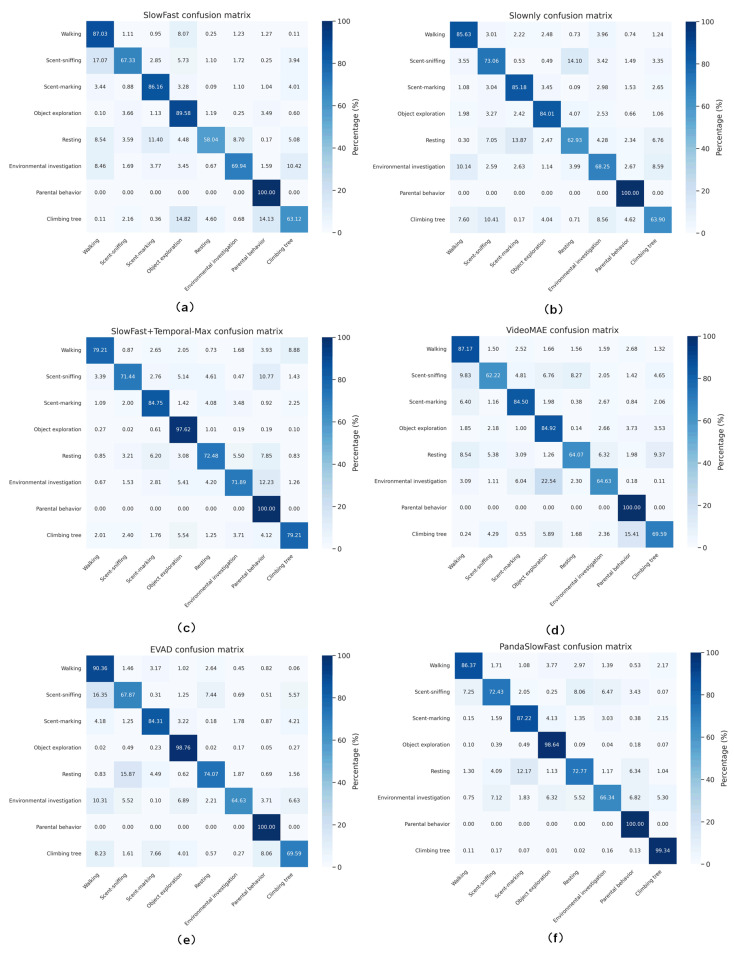
**Confusion matrices of various models.** (**a**) Confusion matrix of SlowFast; (**b**) confusion Matrix SlowOnly; (**c**) confusion matrix of SlowFast + Temporal-Max; (**d**) confusion matrix of VideoMAE; (**e**) confusion matrix of EVAD; (**f**) confusion matrix of PandaSlowFast.

Figure 11 presents the confusion matrices of six different behavior recognition models, providing an intuitive comparison of their performance across multiple behavior categories. Overall, compared to the baseline SlowFast model (a), PandaSlowFast achieves significantly higher precision in critical behaviors such as exploration, sniffing, and scent marking, indicating enhanced spatiotemporal feature discrimination and temporal modeling capacity. In contrast to the SlowOnly model (b), PandaSlowFast not only improves detection accuracy in the above-mentioned behaviors but also shows notable gains in recognizing exploratory and parental behaviors, which are characterized by greater temporal complexity. This suggests the model’s improved ability to capture fine-grained temporal dynamics. Compared to (c) SlowFast + Temporal-Max Pooling, which enhances temporal expressiveness through simple pooling mechanisms, our model achieves better discrimination between semantically similar behaviors such as sniffing and exploration. This highlights the effectiveness of the temporal attention mechanism in reducing confusion among visually and temporally overlapping actions. While recent video representation learning models such as VideoMAE (d) and EVAD (e) show promising performance, they still exhibit lower precision in challenging behavior categories such as sniffing, exploration, and climbing. In contrast, PandaSlowFast delivers consistent improvements across these categories, indicating its robustness in modeling subtle motion cues and spatiotemporal context. Nevertheless, all models show performance drops in classifying sniffing, resting, and exploration behaviors. This may be attributed to the high visual and temporal similarity among these behaviors, which limits the discriminative power of current feature extractors. Moreover, the temporal continuity and ambiguous transitions commonly observed in naturalistic wild scenarios further complicate behavior boundary identification, increasing the overall classification difficulty.

### 3.4. Edge Deployment Performance Analysis

To evaluate the practical applicability of the proposed PandaSlowFast model under resource-constrained conditions, we conducted a comprehensive performance benchmark on an edge device. Both FP32 and FP16 ONNX versions of the model were deployed and compared in terms of detection accuracy, inference latency, throughput, and peak memory usage.

Based on Table 7, the FP32 configuration achieved an mAP of 85.37% but exhibited relatively high inference latency, resulting in a throughput of approximately 1.9 FPS, and therefore serves as a baseline deployment. After FP16 quantization, the model maintained a high mAP of 85.16%, with only a marginal accuracy degradation of 0.21%, while inference latency was reduced to 310 ms per frame, corresponding to a throughput of approximately 3.2 FPS, and peak memory consumption was reduced by about 29%. These results demonstrate that FP16 quantization provides a favorable trade-off between accuracy and efficiency, substantially improving inference speed and resource utilization with negligible performance loss. Consequently, PandaSlowFast can be deployed on low-cost, low-power edge platforms such as Raspberry Pi for near–real-time inference. Given that giant panda behaviors captured by infrared camera traps typically evolve at relatively slow temporal scales, the achieved inference speed is sufficient for practical long-term behavioral monitoring. Future work will further explore model pruning and knowledge distillation to enhance deployment efficiency while preserving recognition accuracy.

## 4. Discussion

The rapid advancement of deep learning technologies has led to an increasing focus among researchers on leveraging these advanced methods for behavior detection studies of giant pandas [9,26]. Accurate, reliable, and cost-effective behavior detection methods are essential for the monitoring and conservation of wild giant pandas [6,38]. This study presents PandaSlowFast, a novel behavior detection framework tailored for wild giant pandas. Compared to previous approaches primarily focused on static behavior classification or captive environments [17], our model introduces key architectural innovations that significantly improve spatiotemporal behavior detection in complex field settings. Specifically, the integration of the TAU attention module enables dynamic adjustment to multiscale temporal cues, enhancing the model’s adaptability across a wide range of behaviors—from transient movements like walking to prolonged states such as resting. Furthermore, the adoption of the ASH activation function addresses the limitations of traditional nonlinearities, improving the model’s ability to capture subtle and nonlinear behavioral variations in unstructured infrared video data.

Compared to earlier approaches constrained by static pose classification [14,16] or limited detection accuracy in complex natural environments [32], the proposed PandaSlowFast model achieves robust and fine-grained behavior detection across multi-view infrared videos of wild giant pandas. This advancement stems not only from architectural improvements—such as the integration of the Temporal Attention Unit (TAU) and the Adaptive SwisH (ASH) activation function—but also from the model’s ability to handle continuous, low-frame-rate video sequences captured in challenging field conditions. These designs enable the model to effectively capture both short-term and long-term behavioral patterns and to identify subtle state transitions, such as the shift from sniffing to scent-marking or from resting to parental care.

The behaviors listed in Table 1 are derived from long-term field monitoring and represent ecologically significant and frequently observed wild giant panda behaviors, including chemical communication, maternal care, and daily activities. These behaviors reflect the diversity of life-history strategies and serve as key indicators of individual health, activity rhythms, and reproductive status [39]. Accurate and consistent monitoring of such behaviors is of great importance for evidence-based conservation and management. Notably, these behaviors often involve subtle, momentary motions or occur under partial occlusion—conditions under which traditional methods often struggle. Our PandaSlowFast model addresses these challenges by leveraging the TAU module to enhance temporal modeling of behavior dynamics and utilizing the ASH activation to improve sensitivity to low-intensity features. This enables the model to reliably detect and distinguish critical behaviors even in visually complex scenarios. By providing frame-level annotations and temporal localization of behaviors in real-world video data, the proposed method offers conservation practitioners a practical tool for real-time assessment of individual well-being, stress responses, reproductive status, and territorial activity. This capability substantially outperforms prior classification-based approaches and lays a scalable foundation for automated, long-term behavioral monitoring of endangered species in the wild.

**Figure 12:**
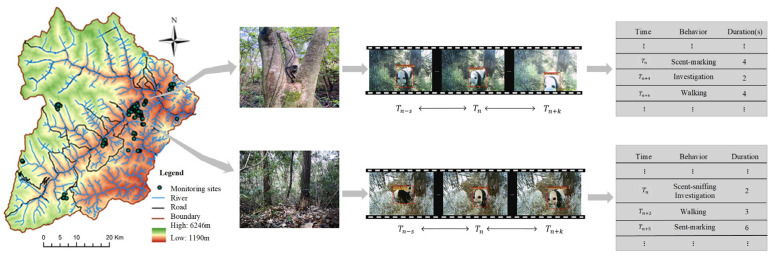
**Conceptual diagram of future applications for giant panda behavior detection models.**

In terms of technical application, our behavior detection algorithm is particularly suitable for resource-constrained edge device applications (e.g., outdoor infrared cameras) and specific target behavior studies (e.g., giant panda monitoring) [6]. The model can integrate geographical and temporal information from videos to comprehensively analyze the occurrence locations and temporal distribution of panda behaviors. For instance, it can associate foraging behavior with specific areas and time periods, aiding researchers in understanding activity patterns, habitat requirements, and behavioral characteristics. In nature reserves, the system supports long-term data accumulation and real-time analysis, assisting decision-makers in optimizing conservation measures. Future applications could include real-time alerts for abnormal behaviors, facilitating rapid responses and offering stronger technical support for wildlife conservation and ecology research [40]. In subsequent work, managers can personalize the integration of the PandaSlowFast model into a comprehensive platform based on research and management needs, enabling the presentation of panda behavior monitoring results in charts, video playback, and other formats. This platform, used in conjunction with infrared cameras, minimizes manual intervention interference and allows real-time data analysis and recording in complex field environments, particularly aligning with China’s ongoing development of a broad scale infrared camera monitoring system for wildlife [41]. This approach enhances the precision of capturing and analyzing the natural behaviors of giant pandas (Figure 12).

To enhance the effectiveness of wild giant panda behavior monitoring using infrared cameras, a series of meticulous deployment strategies and technical measures can be implemented in the field. For instance, infrared cameras should be installed along the animals’ selected pathways, ensuring their height aligns with the typical activity level of giant pandas. Multiple infrared cameras can be positioned at a single deployment site to capture activities from various angles, with a particular emphasis on obtaining side-profile videos. Side views provide clearer representations of behavioral characteristics, avoiding issues such as facial obstruction or motion restrictions common in frontal shots. Additionally, considering the complex lighting conditions of the field environment, researchers should select high-performance infrared camera equipment with high adaptability, ensuring stable operation in low-light and high-contrast environments to capture clear and accurate images.

Based on our research, several important considerations for analyzing behavioral information and constructing models after collecting field monitoring data include: (1) The establishment of a behavioral dataset is crucial, involving data collection, preprocessing, and annotation. Confusion matrix results indicate that low recognition accuracy for some behaviors is primarily due to the similarity in postures and actions among different behaviors, leading to potential recognition biases. Future research should focus on improving accuracy in detecting similar behavioral data. (2) During data preprocessing, researchers need to select a diverse training set that includes images of giant pandas exhibiting various postures, environments, and behaviors. Video data processing often requires consideration of aspect ratio issues to avoid image distortion, necessitating necessary cropping or edge extension in early data protocols. (3) Once data preprocessing is completed, researchers can utilize deep learning models for automated behavior annotation, generating an initial behavioral dataset. For detecting behaviors across multiple individuals, instance segmentation methods are particularly effective, especially in scenarios with multiple giant pandas in the wild. Advanced segmentation networks like U-Net [42] have demonstrated considerable potential in this task, effectively facilitating feature extraction and instance segmentation of giant panda behaviors.

The integration of infrared cameras and deep learning technologies shows significant application potential for application in the intelligent behavioral detection of globally rare and endangered wildlife. Taking felids and primates as examples, the habitats of top predators such as snow leopards and tigers within the felid family are more covert, and current monitoring efforts heavily rely on infrared cameras, making the analysis of behavioral data crucial. The model’s adaptability to various environmental conditions and its capability to process multi-view video data make it more aligned with the actual monitoring scenarios of these animals. Additionally, the lightweight design of the model facilitates its deployment on field infrared camera monitoring platforms. For another major group of mammals—primates—their behavioral manifestations are more complex, exhibiting higher-order and more intricate behavioral categories, such as multimodal communication behaviors and social behaviors. This complexity poses greater challenges for manual analysis, leading to more pronounced analysis errors. Traditional behavior recognition models generally lack the ability to capture and analyze temporal and spatial dynamics effectively. The PandaSlowFast model, however, can be adjusted to monitor and analyze the behaviors of these species with high precision and efficiency, particularly advantageous in studying social interactions and individual behaviors among primates.

The proposed model still presents several limitations. PandaSlowFast is primarily trained and validated on datasets of wild giant pandas and has not yet been systematically evaluated for cross-species generalization. As a result, its accuracy may decline when applied to species with markedly different body sizes, behavioral patterns, or motion characteristics, requiring additional fine-tuning or transfer learning. Meanwhile, our sampling strategy may result in certain rare behaviors—such as high-altitude movements, behaviors occurring under extreme environmental conditions, or reproduction-related activities—being underrepresented in the dataset, which may impose potential limitations on the overall generalization capacity of the AI models. Furthermore, the model relies on the quality and diversity of annotated data. Yet, infrared camera footage from the field is often affected by fixed viewpoints, complex lighting conditions, and occlusions, which can lead to domain shifts when deployed in different reserves or with varying devices. Although PandaSlowFast is relatively lightweight compared to Transformer-based architectures, achieving real-time inference on edge devices such as field camera traps still requires further optimization through model pruning or knowledge distillation. This study targets the vulnerable wild giant panda, with data collected from the remote high-altitude forests of the Wolong Nature Reserve in Sichuan, China. Due to the species’ rarity, elusive behavior, and harsh field conditions, data collection is extremely challenging, resulting in a relatively small dataset despite years of effort. This limitation reflects a broader challenge in intelligent wildlife monitoring, where large-scale data for rare species are inherently difficult to obtain. Future research will focus on cross-species validation, domain adaptation, and model lightweighting to enhance its generalizability and practical deployability.

In resource-constrained application scenarios, improving model inference efficiency while maintaining detection accuracy remains a key research challenge in video behavior detection. Recent studies have proposed a range of lightweight architectures that demonstrate promising performance. For example, MobileNetV3 [43] enhances computational efficiency through structural compression and channel pruning; the Temporal Shift Module (TSM) introduces temporal modeling capabilities while maintaining a compact structure; and X3D [8] achieves a favorable trade-off between speed and accuracy. These approaches have shown competitive results across various tasks and datasets, indicating their generalizability and offering valuable design insights for lightweight models under limited computational resources. Therefore, while this work focuses on the accuracy-first PandaSlowFast framework, we also suggest that future research may explore integrating such efficient architectures—via module replacement or pruning—into behavior detection systems to enhance deployability on edge devices.

In summary, the vast amounts of animal monitoring images and videos provide rich data for tracking the behaviors of target individuals. This technology not only helps us better understand the behavioral patterns of pandas but also offers methodological support for the behavioral studies of other rare and endangered species.

## 5. Conclusions

This study addressed the challenges of low data utilization and the difficulty of detection technologies in adapting to complex field environments for wild giant panda behavior monitoring. We constructed a wild giant panda behavior detection dataset based on long-term infrared camera monitoring and proposed an improved PandaSlowFast intelligent behavior detection model. By introducing a temporal attention mechanism and an adaptive activation function, the model effectively enhances spatiotemporal feature extraction capabilities in complex natural environments, achieving 85.38% mean average precision on real-world field datasets—significantly outperforming existing methods. Furthermore, the quantized lightweight model achieved real-time deployment on edge devices such as the Raspberry Pi, validating its feasibility and application potential in practical conservation work. Based on integrated estimates from molecular biological analysis and field monitoring records, this study covered at least 20 individual wild giant pandas. Habitat analysis further revealed that pandas prefer coniferous–broadleaf mixed forests at elevations of 1600–3400 m, characterized by gentle terrain, accessible water sources, and abundant staple bamboo, while clearly avoiding areas with strong human disturbance. These findings provide important scientific evidence for precise giant panda habitat management and ecological corridor planning. The technical framework proposed in this study demonstrates strong transferability, laying a methodological foundation for future expansion to intelligent monitoring and conservation research on other rare species and thereby strongly supporting the digital transformation of biodiversity conservation.

## Figures and Tables

**Table 1:** **Definitions of giant panda behaviors in the wild.**

Behavior Name	Definition
Walking	The giant panda moves across the field of view of the infrared camera, exhibiting significant relative displacement in the video.
Scent-marking	The giant panda applies secretions from its anal glands or urine onto a tree trunk. This behavior primarily occurs with the hind limbs and typically involves interaction with the trunk or vegetation.
Scent-sniffing	Corresponding to scent-marking, the giant panda uses its mouth or nose to sniff a tree trunk or vegetation, usually interacting with these objects.
Object exploration	The giant panda engages in tactile exploration of interesting objects (such as artificially installed cameras or branches found in the wild) using its mouth or nose. This behavior primarily occurs with the head and generally involves interaction with the objects of interest.
Resting	The giant panda remains stationary, either standing or lying on the ground for an extended period, or displays evident behaviors of lying down or reclining.
Environmental investigation	The giant panda maintains a short period of stillness, often accompanied by scanning its surroundings visually to gather environmental information. After assessing the obtained information, it will typically engage in subsequent behaviors.
Parental behavior	This behavior typically occurs between adult female pandas and their offspring, characterized by interactive behaviors aimed at enhancing the survival of the young.
Climbing tree	The giant panda wraps its limbs around a large tree trunk. This behavior is usually performed quickly, lasting a relatively short time, and involves interaction with the trunk.

**Table 2:** **Benchmark performance test of the wild giant panda behavior detection algorithm.**

Model Name	Parameters (M)	mAP (%)
SlowFast + CvT + AdamW [35]	-	46.2
ACRN [19]	-	76.79
SlowFast [27]	34.6	77.65
SlowFast + Temporal-Max [27]	34.6	83.15
VideoMAE [37]	129.3	77.14
EVAD [32]	186.4	82.67
PandaSlowFast (Ours)	35.2	85.37

**Table 3:** **Results of ablation experiments for the wild giant panda behavior detection model.**

Adaptive SwisH	Temporal Attentional	mAP
		83.15%
√		83.95% (+0.80%)
	√	85.16% (+2.01%)
√	√	85.37% (+2.22%)

**Table 4:** **Experimental results of different sampling rates in the Fast pathway.**

	mAP	GFLOPs
SlowOnly	81.64%	27.3
β = 1/4	83.43%	54.5
β = 1/6	84.69%	41.8
β = 1/8	85.37%	36.1
β = 1/12	84.52%	32.8
β = 1/16	84.31%	30.6

**Table 5:** **Statistical reliability verification based on three independent training runs.**

Model	Trial 1 (mAP %)	Trial 2 (mAP %)	Trial 3 (mAP %)	Mean ± Std (mAP %)
SlowFast (Baseline)	83.15	83.20	83.17	83.17 ± 0.03
+ASH	83.95	83.91	83.98	83.95 ± 0.04
+TAU	85.16	85.12	85.19	85.16 ± 0.04
PandaSlowFast	85.37	85.35	85.43	85.38 ± 0.04

**Table 6:** **Impact of the TAU module on short- and long-duration behavior detection (mAP, %).**

Model	Short-Duration (<2 s)	Long-Duration (>5 s)	Overall
SlowFast (Baseline)	80.1	82.5	83.15
PandaSlowFast (Ours)	81.0	85.7	85.37
Gain	+0.9	+3.2	+2.22

**Table 7:** **Edge deployment performance benchmark of PandaSlowFast.**

Model Configuration	mAP (%)	Inference Latency (ms/Frame)	FPS	Peak Memory (MB)
PandaSlowFast (FP32 ONNX)	85.37	520	~1.9	680
PandaSlowFast (FP16 ONNX)	85.16 (−0.21)	310	~3.2	480

## Data Availability

Code used for the AI model is available from this link: https://pan.nwafu.edu.cn/share/08544462ff443b3001d50833de (accessed on 15 March 2026).
